# Characterization and Potential Application of Phage vB_PmuM_CFP3 for Phage Therapy Against Avian *Pasteurella multocida*

**DOI:** 10.3390/ani14223268

**Published:** 2024-11-13

**Authors:** Hongmei Chen, Nansong Jiang, Guanghua Fu, Qiuling Fu, Chunhe Wan, Yu Huang, Yuan Liu, Rongchang Liu, Qizhang Liang, Longfei Cheng

**Affiliations:** 1 Institute of Animal Husbandry and Veterinary Medicine, Fujian Academy of Agricultural Sciences, Fuzhou 350013, China; chenhmei052@126.com (H.C.); nansongjiang@126.com (N.J.); fuyuan163@163.com (G.F.); qiulingfu0822@163.com (Q.F.); chunhewan@126.com (C.W.); huangyu_815@163.com (Y.H.); liurongc@foxmail.com (R.L.); 11817043@zju.edu.cn (Q.L.); 2 Fujian Industry Technology Innovation Research Academy of Livestock and Poultry Diseases Prevention and Control, Fuzhou 350013, China; 3 Fujian Key Laboratory for Control and Prevention of Avian Diseases, Fuzhou 350013, China; 4 College of Veterinary Medicine, Yangzhou University, Yangzhou 225009, China; liuyuan2018@yzu.edu.cn

**Keywords:** phage CFP3, avian cholera, *Pasteurella multocida*, phage therapy, antibiotic alternative

## Abstract

The rise of antibiotic-resistant bacterial infections necessitates alternative therapeutic strategies, such as phage therapy. This study investigates the potential of phage vB_PmuM_CFP3 (CFP3) as a therapeutic agent against avian cholera caused by *Pasteurella multocida* (*P. multocida*). Phage CFP3 was isolated from the feces and wastewater of a laying hen farm and underwent comprehensive biological characterization, including host range, lytic activity, and environmental stability. Transmission electron microscopy revealed CFP3′s typical myovirus morphology, with a head diameter of approximately 60 nm and a tail length of about 120 nm. CFP3 demonstrated high stability across a pH range of 4–10 and temperatures of 30–40 °C, making it suitable for oral administration in poultry. The phage exhibited a latent period of about 90 min and an optimal multiplicity of infection (MOI) of 1. Despite its narrow host range, with a lysis rate of 28.2% against avian-derived type A *P. multocida*, CFP3′s specificity minimizes impact on non-target bacteria. Whole-genome sequencing revealed a 32,696 bp linear double-stranded DNA genome with 46 predicted open reading frames (ORFs) and no tRNA or antibiotic resistance genes, enhancing its safety profile. Phylogenetic analysis indicated a close evolutionary relationship with *Haemophilus* phages HP1, HP2, and *Pasteurella* phage F108. While CFP3 shows promise as a precision therapeutic tool, further in vivo studies are required to evaluate its efficacy and safety. Future research should focus on expanding the phage library, optimizing phage mixtures, and exploring synergistic effects with other antimicrobial strategies. This study provides foundational data supporting the development of CFP3 as a viable alternative to antibiotics for controlling avian cholera.

## Simple Summary

Phage therapy offers a targeted approach to combat antibiotic-resistant infections. In this study, we isolated a phage named vB_PmuM_CFP3 (CFP3) from a chicken farm to specifically target *Pasteurella multocida*, the bacterium responsible for avian cholera. Our research showed that CFP3 effectively attacks the bacteria while being stable under conditions suitable for poultry treatment. Safety checks revealed that CFP3 lacks genes related to antibiotic resistance, making it a safer option. Our findings suggest that CFP3 could be a viable alternative treatment for birds without harming other beneficial bacteria. While the initial findings are promising, further studies are needed to confirm these results in living animals. Our work provides an important step towards using phage therapy for safer, sustainable management of avian cholera.

## 1. Introduction

*Pasteurella multocida* (*P. multocida*) is a non-motile, facultatively anaerobic, Gram-negative coccobacillus that poses a significant threat to a wide range of domestic and wild mammals, birds, reptiles, and humans [1,2,3]. This versatile pathogen is classified into five capsular types: A, B, D, E, and F. Each is associated with different host species and disease manifestations [4]. Among these, capsular type A *P. multocida* is the causative agent of avian cholera, a highly contagious and economically devastating disease in the poultry industry [5,6,7,8]. Avian cholera caused by *Pasteurella multocida* represents a significant economic burden for the poultry industry, resulting in substantial losses due to high morbidity and mortality rates among infected flocks.

The primary strategies for controlling avian cholera include vaccination and antibiotic treatment [9,10]. Currently available commercial vaccines, including both live attenuated and inactivated types, provide only moderate levels of protection. The efficacy of these vaccines can be variable, and they may not offer complete immunity, necessitating the need for booster doses and additional biosecurity measures. Furthermore, the widespread use of antibiotics has led to the emergence of antibiotic-resistant strains of *P. multocida* complicating treatment efforts and prompting regulatory agencies to advocate for reduced and restricted antibiotic use in agriculture. In light of these challenges, there has been a resurgence of interest in phage research as an alternative approach to bacterial disease prevention and treatment. Phages are viruses that specifically infect and lyse bacteria [10]. They offer several advantages over traditional antibiotics, including specificity for their host bacteria, the ability to disrupt biofilms, and a lower likelihood of inducing resistance mutations. *P. multocida* phages have been extensively studied as a potential alternative to traditional antibiotics for controlling avian cholera [11]. 

Researchers have successfully isolated and characterized a diverse array of phages that specifically target *P. multocida* strains associated with poultry infections. These phages have demonstrated promising capabilities in combating *P. multocida* infections. Studies have shown that phages can effectively lyse and kill *P. multocida* cells, disrupting the formation of bacterial biofilms that protect the pathogen from host immune responses and antimicrobial agents [12,13,14]. Additionally, phage lysins, the enzymes responsible for degrading the bacterial cell wall, have been investigated for their potential as stand-alone antimicrobial agents [15,16].

This study aims to explore the potential of phages as a therapeutic tool against avian cholera. We isolated phages targeting avian *P. multocida* from the feces and wastewater of a laying hen farm. Following isolation, we conducted a comprehensive biological characterization of the phages, including their host range, lytic activity, and stability under various environmental conditions. Additionally, whole-genome sequencing was performed to elucidate the genetic makeup of the isolated phages, providing insights into their mechanisms of action and potential for therapeutic application.

## 2. Materials and Methods

### 2.1. Main Reagents and Preparation

Martin agar and Martin broth were purchased from Qingdao Hi-Tech Industrial Park Haibo Biotechnology (Qingdao, China) Co., Ltd. The upper layer of Martin agar was prepared by halving the dry powder according to the Martin agar formula, and 2× Martin broth was prepared by doubling the dry powder amount according to the formula. Agar, chloroform, calcium chloride, sodium chloride, magnesium sulfate, and 1 mol/L Tris-HCl were purchased from Sangon Biotech (Shanghai, China) Co., Ltd. The composition of SM buffer was 100 mmol/L NaCl, 10 mmol/L MgSO_4_·7H_2_O, and 50 mmol/L Tris-HCl, with the pH adjusted to 7.5. It was sterilized by autoclaving and stored at room temperature. The 220 nm syringe filters were purchased from Merck Millipore Ltd. (Tullagreen, Carrigtwohill, CO Cork, Ireland).

### 2.2. Bacterial Strains and Culture Methods

*P. multocida* CVCC44801 (capsular type A) and *P. multocida* CVCC392 (capsular type D) were obtained from the China Veterinary Culture Collection Center. Thirty-nine avian *P. multocida* isolates (capsular type A), and *P. multocida* FCf79 (capsular type F) from monkeys were preserved at the Institute of Animal Husbandry and Veterinary Medicine, Fujian Academy of Agricultural Sciences. The bacterial strains were revived from lyophilized powder by streaking on Martin agar and incubating at 37 °C for 20 h. Two to three typical colonies were then inoculated into 20 mL of Martin broth and incubated at 37 °C for 14–18 h. The cultures were stored at 2–8 °C and used within 5 days. The phage titers were determined by diluting the phage suspension using SM buffer in a series of tenfold dilutions. An appropriate volume of each dilution was mixed with the host bacteria and spotted on double-layer agar plates. The plaques formed were counted, and the phage titer was calculated based on the number of plaques on plates with the appropriate density. The results are expressed as PFU/mL, indicating the number of plaque-forming units per milliliter of phage suspension.

### 2.3. Isolation and Purification of Avian P. multocida Phages

The double-layer agar method with enrichment was used. Approximately 100 g of fresh feces were collected from a laying hen farm and added to 400 mL of SM buffer. Additionally, 500 mL of wastewater from the farm was collected, and calcium chloride was added to a final concentration of 1.25 mmol/L. The samples were mixed by inversion and left to stand, then filtered through gauze. The filtrate was centrifuged at 10,000× *g* for 10 min, and the supernatant was filtered through a 220 nm syringe filter. The filtrate was mixed with an equal volume of 2× Martin broth, and 0.5% (*v*/*v*) of fresh bacterial cultures (strains FCf16, FCf45, FCf53, FCf62, FCf91, and FCf98) were added. The mixture was incubated overnight at 37 °C. The culture was centrifuged again, and the process was repeated with fresh cultures, incubating overnight at 37 °C. Five milliliters of the culture were then mixed with 50 μL of chloroform, inverted to mix, and left to stand. The mixture was centrifuged at 10,000× *g* for 10 min, and the supernatant was filtered through a 220 nm syringe filter. One hundred microliters of the filtrate were mixed with each of the six bacterial strains and added to the upper layer of Martin agar in a 50 °C water bath, then poured onto Martin agar plates. After natural cooling and solidification, the plates were incubated overnight at 37 °C. Single plaques were picked from the plates and soaked in 1 mL of SM buffer for several hours. The supernatant was filtered through a 220 nm syringe filter to obtain the phage solution. This purification process through plaque picking and re-isolation was repeated six times to obtain purified phages.

### 2.4. Electron Microscopy Observation of Phages

A small amount of phage solution was placed onto a copper grid and left for 15 min. The liquid was then wicked off from the side, and a drop of 20 g/L phosphotungstic acid (pH 6.8) was added to the grid for staining. After allowing the stain to act for 5 min, the liquid was wicked off again from the side, and the grid was dried. The sample was observed using a Hitachi HT7700 transmission electron microscope (Hitachi High-Technologies Corporation, Ibarakiken, Japan).

### 2.5. Thermal Stability Test of Phage CFP3

The phage was adjusted to a titer of approximately 5 × 10^7^ PFU/mL and aliquoted into 24 EP tubes, each containing 1 mL. The tubes were incubated in water baths at 30 °C, 40 °C, 50 °C, and 60 °C. At 30 min and 60 min intervals, three tubes were removed from each temperature and immediately placed in an ice-water bath for 5 min. The phage titers were then measured to analyze the thermal stability of the phage.

### 2.6. PH Stability Test of Phage CFP3

The phage was adjusted to a titer of approximately 5 × 10^7^ PFU/mL and aliquoted into 10 EP tubes, each containing 100 μL. To each tube, 900 μL of SM buffer with pH values of 3.0, 4.0, 5.0, 6.0, 7.0, 8.0, 9.0, 10.0, 11.0, and 12.0 was added. The tubes were incubated in a water bath at 25 °C for 1 h, and the phage titers were measured. The experiment was repeated three times to analyze the pH stability of the phage.

### 2.7. Determination of Optimal Multiplicity of Infection (MOI) for Phage CFP3

FCf91 bacterial culture was counted and diluted with Martin broth. The bacteria and phage were mixed at MOI ratios (phage PFU to bacterial CFU) of 10, 1, 0.1, 0.01, and 0.001. Each mixture contained 100 μL of bacterial culture and an equal volume of phage solution. The mixtures were incubated in a water bath at 37 °C for 10 min; then, Martin broth was added to bring the total volume to 20 mL. The cultures were incubated at 37 °C with shaking at 200 r/min for 5 h. One milliliter of the culture was then mixed with 10 μL of chloroform, centrifuged at 10,000× *g* for 5 min at 4 °C, and the supernatant was collected to measure the phage titer. The MOI corresponding to the highest phage titer was considered the optimal MOI. The experiment was repeated three times.

### 2.8. One-Step Growth Curve of Phage CFP3

The phage CFP3 was adjusted to a titer of approximately 5 × 10^7^ PFU/mL. One hundred microliters of the phage were mixed with an equal volume of FCf91 host bacteria at an MOI of 1. The mixture was incubated in a water bath at 37 °C for 10 min, then centrifuged at 10,000× *g* for 3 min at 4 °C. The supernatant was discarded, and the pellet was resuspended in 20 mL of TSB pre-warmed in a 37 °C water bath. The culture was incubated at 37 °C with shaking at 200 r/min. Samples of 200 μL were taken at time zero and every 15 min thereafter, up to 2 h. The samples were centrifuged at 10,000× *g* for 3 min at 4 °C, and the supernatant was collected to measure the phage titer. The experiment was repeated three times. A one-step growth curve was plotted with the infection time on the *x*-axis and the logarithm of the phage titer on the *y*-axis.

### 2.9. Lysis Spectrum of Phage CFP3

The phage solution was diluted to approximately 1 × 10^4^ PFU/mL. One hundred microliters of the diluted phage solution were mixed with an equal volume of each bacterial strain. After standing for 15 min, 5 mL of top-layer Martin agar pre-warmed at 50 °C was added to the mixture and poured onto Martin agar plates. The plates were left to solidify for 2 h and then incubated at 37 °C overnight. The presence of plaques on the plates indicated that the corresponding bacterial strain could be lysed by the phage; otherwise, it was considered non-lytic.

### 2.10. Whole Genome Sequencing and Analysis of Phage CFP3

The whole-genome sequencing of the phage was outsourced to Shanghai LingEn Biotechnology (Shanghai, China) Co., Ltd.

### 2.11. Sequencing

The phage genome was extracted, and a library was constructed using the Illumina TruSeqTM Nano DNA Sample Prep Kit (San Diego, CA, USA). After quantification, the library was mixed according to the data ratio and sequenced on the Illumina NovaSeq6000 platform to obtain read sequences. Low-quality sequencing data whose sequencing quality value was less than Q20 and small fragments whose length was less than 75 bp after quality pruning were filtered to obtain high-quality clean data by Trimmomatic (v0.35) software.

### 2.12. Assembly

The assembly software ABySS (v2.1.5) was used to perform assembly with multiple Kmer parameters to obtain the optimal assembly result (http://www.bcgsc.ca/platform/bioinfo/software/abyss; accessed on 6 April 2024). GapCloser software (https://sourceforge.net/projects/soapdenovo2/files/GapCloser/; accessed on 8 April 2024) was then used for local gap filling and base correction to obtain the genome sequence.

### 2.13. Assembly Result Evaluation

The reads were aligned to the assembled genome sequence, and the GC content and coverage depth of the assembled sequences were calculated. The GC-depth distribution showed a Poisson distribution, and the overall GC content distribution and sequencing coverage of the assembled sequences were normal.

### 2.14. Prediction of Genome Coding Genes

The software GeneMarkS (http://topaz.gatech.edu/GeneMark/; version 4.28; accessed on 7 April 2024) was used to predict coding genes in the genome. The predicted protein sequences were compared with the NR, Swiss-Prot, eggNOG, KEGG, and GO databases to obtain annotation information for the predicted genes. Finally, the genomic information was visualized by the online software Proksee (https://proksee.ca/; accessed on 8 April 2024).

### 2.15. Construction of Phylogenetic Tree and Genomic Synteny Analysis

The phylogenetic tree based on the whole genome sequence was compared by the online platform PhageScope (https://phagescope.deepomics.org/; accessed on 12 April 2024), and the phylogenetic tree was constructed by the “Comparative Tree Construction” function. Based on the amino acid sequence, the Neighbor-joining method was used to construct the evolutionary tree through the Muscle package alignment in MEGA-X, in which the bootstrap with 1000. The resulting phylogenetic tree can be enhanced and visualized using the iTOL website (https://itol.embl.de/; accessed on 12 April 2024). The genomic synteny comparison at the nucleotide level was generated via Easyfig (v2.2.5).

## 3. Results

### 3.1. Isolation and Electron Microscopy Morphology of the Phage

**Figure 1:**
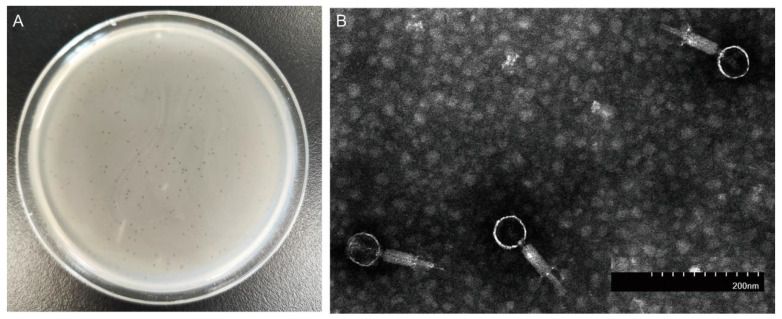
Isolation and electron microscopy morphology of phage. (**A**) Plate image of avian *P. multocida* phage vB_PmuM_CFP3; (**B**) Electron micrograph of phage vB_PmuM_CFP3.

This study successfully isolated a phage capable of lysing FCf91 by processing and double enriching samples from a chicken farm’s feces and sewage and then cultivating them with FCf91 bacterial culture on double-layer agar plates. Clear plaques were observed on the plates, which are transparent regions formed after the phage infected and lysed the bacteria (Figure 1A). These plaques varied in size but were evenly distributed, indicating that the phage effectively infects and lyses bacteria on the plates. The presence of these plaques demonstrates that this phage has strong lytic activity and can effectively infect and kill *P. multocida*. Subsequently, negative staining and transmission electron microscopy (TEM) were used to observe the phage (Figure 1B), clearly revealing the detailed structure of the phage particles. The phage consists of a nearly spherical head and a tail. The head has a cross-sectional diameter of approximately 60 nanometers and presents a polyhedral structure that likely contains the phage’s genetic material. The tail is longer, about 120 nanometers, with a contractile sheath structure likely used for attaching to and penetrating the host bacterial cell wall. Based on its morphological characteristics, the phage was named vB_PmuM_CFP3, abbreviated as CFP3.

### 3.2. Stability and Growth Characteristics of Phage CFP3

**Figure 2:**
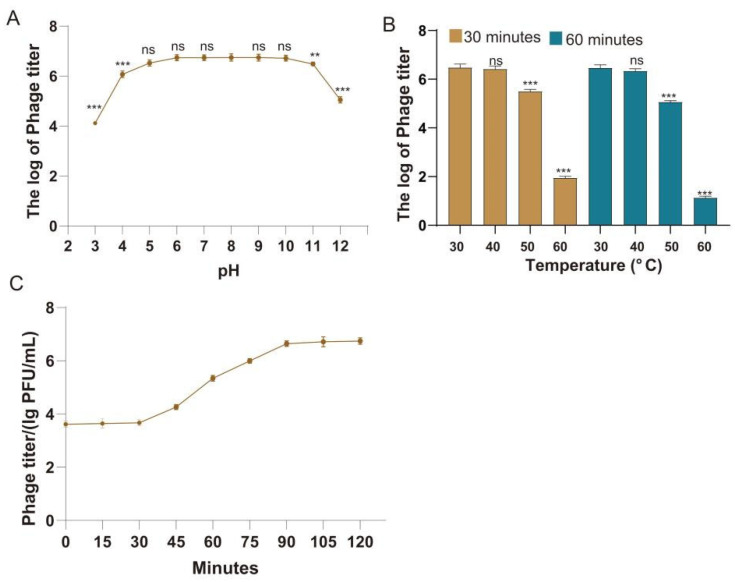
Stability and growth characteristics analysis of phage CFP3. (**A**) Results of pH stability test of phage CFP3; (**B**) Results of thermal stability test of phage CFP3; (**C**) Growth curve of phage CFP3. ns, *p* > 0.05; **, *p* < 0.01; ***, *p* < 0.001.

The phage titer logarithm of phage CFP3 under varying pH conditions reveals that the phage maintains high stability across pH values ranging from 5 to 10 (Figure 2A). Within this range, the phage titer remains consistently elevated, with an optimal peak observed at a pH of 8. This suggests that phage CFP3 exhibits the greatest stability under neutral to mildly alkaline conditions. In contrast, when the pH falls below 4 or exceeds 11, the phage titer sharply declines, indicating that extremely acidic or alkaline conditions negatively impact the phage’s stability.

Thermal stability tests for phage CFP3 at various temperatures show that after 30 and 60 min of exposure at 30 °C and 40 °C, the logarithm of the phage titer remains relatively unchanged, denoting good thermal stability at these temperatures. Conversely, a significant decrease in phage titer is observed as the temperature rises to 50 °C and 60 °C. At 60 °C, particularly after 60 min, the phage titer nearly drops to its lowest level, suggesting that high temperatures significantly compromise the stability of phage CFP3 (Figure 2B).

The growth curve of phage CFP3 indicates a slow increase in phage titer during the first 30 min, followed by a rapid escalation between 30 and 90 min, reaching its peak. After 90 min, the growth of the phage titer plateaus, indicating that the phage has reached a stable growth state (Figure 2C).

In summary, phage CFP3 demonstrates excellent pH stability under neutral to mildly alkaline conditions, substantial thermal stability at 30 °C and 40 °C, and achieves a stable growth phase after 90 min of cultivation.

### 3.3. Optimal Multiplicity of Infection (MOI) for Phage CFP3

To determine the optimal multiplicity of infection (MOI) for phage CFP3 infecting its host bacterium FCf91, a series of experiments were conducted under varying MOI conditions. Phages and bacteria were mixed at different MOI ratios and incubated at 37 °C with shaking at 200 r/min for 5 h. After the incubation period, the phage titers in each culture were measured. Table 1 presents the initial phage count, bacterial count, and final phage titer for phage CFP3 under different MOI conditions. The experimental results indicate that at a MOI of 1, the phage titer in the culture reached its highest level, measured at (6.9 ± 0.5) × 10^8^ PFU/mL. This finding suggests that a MOI of 1 is the most efficient condition for phage CFP3 to infect its host bacterium, FCf91.

### 3.4. Lytic Spectrum of Phage CFP3

The lytic spectrum of phage CFP3, as detailed in Table 2, demonstrates its varied lytic capabilities against *P. multocida* strains from different sources and capsular groups. Specifically, phage CFP3 was unable to lyse *P. multocida* strains from capsular group D (bovine source) and capsular group F (monkey source), indicating a lack of lytic activity against these groups. However, out of 39 avian-derived strains from capsular group A, phage CFP3 was able to lyse 11 strains, resulting in a lysis rate of 28.2%. This suggests that while phage CFP3 possesses some lytic activity against capsular group A strains of *P. multocida*, this activity is highly specific and only effective against certain strains.

The detailed lysis patterns of the strains are presented in Table 2, showing varied results for strains from chickens, ducks, and geese within capsular group A. For instance, phage CFP3 was able to lyse chicken-derived strains such as FCf93, FCf58, and FCf65, but it could not lyse strains like FCf88, FCf94, and FCf95. In summary, phage CFP3 exhibits high specificity by lysing only certain strains within capsular group A of *P. multocida*. These findings provide valuable insights into the potential application of phage CFP3 for the targeted control of specific pathogenic strains.

### 3.5. Genomic Sequencing Results of Phage CFP3

**Figure 3:**
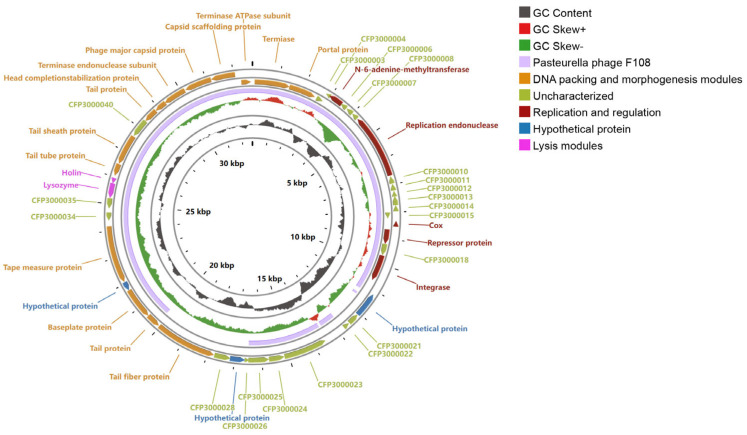
Genomic sequencing results of phage CFP3. The figure displays the circular map of the genomic structure of phage CFP3, showing the GC content and GC skew in the outer ring. Different colored bands represent functional modules, including DNA packaging and morphogenesis, replication and regulation, structure and assembly, and lysis modules, among others.

Figure 3 illustrates the circular map of the CFP3 genome, with the outer ring indicating GC content and GC skew. Different colored bands in the figure represent various functional modules, including DNA packaging and morphogenesis, replication and regulation, structure and packaging, and lysis modules. The whole-genome sequencing results reveal that the genome of phage CFP3 is a linear double-stranded DNA. The genome’s total length is 32,696 bp, with a G + C content of 41.7%. The genome contains no tRNA genes or antibiotic-resistance genes. The genome comprises 46 genes (open reading frames, ORFs), as shown in Table 3. Among these, 21 are known to encode functional proteins, including capsid proteins (e.g., ORF44 and ORF45), head structure proteins (e.g., ORF42), tail structure proteins (e.g., ORF29, ORF30, ORF38, ORF39, and ORF41), lysis-related proteins (e.g., ORF36 and ORF37), and metabolic and regulatory proteins (e.g., ORF9 and ORF17). The total length of these genes is 30,579 bp, accounting for 93.5% of the genome, with an average gene length of 664 bp, indicating high gene density. The genome sequence has been submitted to GenBank with the accession number OQ025560. Detailed analysis of the CFP3 genome provides essential insights into its genetic composition and function, laying a critical foundation for further studies on its biological characteristics and application potential.

### 3.6. Phylogenetic Analysis of Phage CFP3

**Figure 4:**
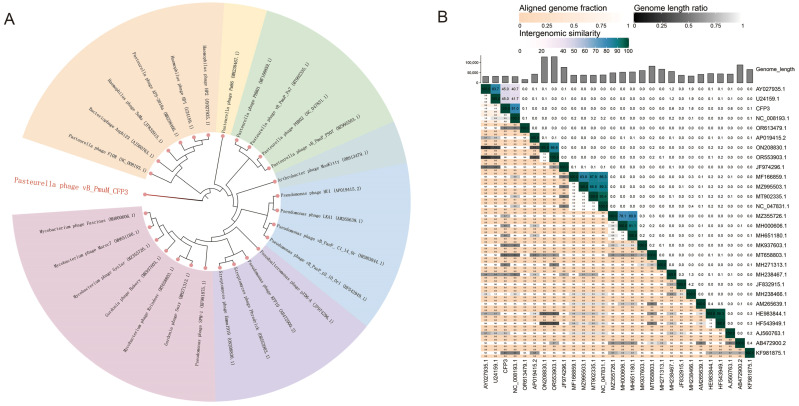
Phylogenetic tree of phage CFP3 based on whole genome sequence. (**A**) A phylogenetic tree of phage CFP3 based on whole genome sequences, demonstrating its evolutionary relationships with other related phages. Different branches in the phylogenetic tree are colored to represent different phage clades, clearly showing the position of phage CFP3 in the evolutionary tree. (**B**) The genomic similarity between phage CFP3 and other phages was calculated using the online tool VIRIDIC (http://rhea.icbm.uni-oldenburg.de/VIRIDIC/; accessed on 14 April 2024). Each square’s color represents the degree of similarity between the two phage genomes, with darker colors indicating higher similarity. The bar chart atop shows the genome length ratio for each phage, with the *x*-axis indicating different phages and the *y*-axis indicating the genome length ratio.

Based on the whole genome sequence, a phylogenetic tree of phage CFP3 was constructed to show its evolutionary relationships with other related phages (Figure 4A). The phylogenetic tree uses different colored branches to represent different phage clusters, clearly displaying the position of phage CFP3 within the tree. It can be seen that phage CFP3 is located in the red branch, showing a close evolutionary relationship with *Haemophilus* phage HP1, *Haemophilus* phage HP2, and *Pasteurella* phage F108, indicating that they share a common ancestor in their evolutionary history. Figure 4B shows the genomic similarity and genome length ratio between phage CFP3 and other phages. Each box in the figure represents the degree of genomic similarity between two phage genomes, with darker colors indicating higher similarity. The bar chart above shows the genome length ratio of each phage, with the *x*-axis representing different phages and the *y*-axis representing genome length ratios. From Figure 4B, it can be seen that phage CFP3 has a high genomic similarity with *Haemophilus* phage HP1, *Haemophilus* phage HP2, and *Pasteurella* phage F108, further supporting the evolutionary relationships demonstrated in the phylogenetic tree.

Overall, these figures provide important insights into the position of phage CFP3 in the phylogenetic tree and its genomic similarity with other phages, offering valuable reference points for understanding the evolutionary history and genomic characteristics of phage CFP3.

### 3.7. Phylogenetic Relationship Analysis of Phage CFP3 Based on Protein Sequences

**Figure 5:**
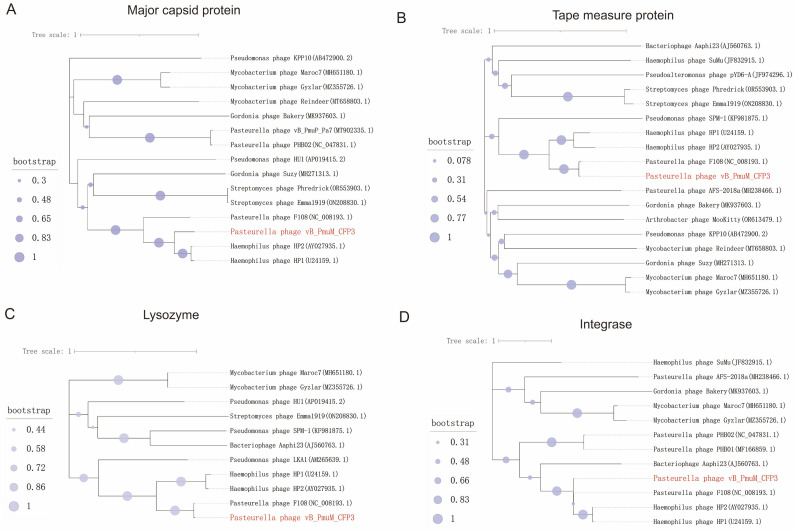
Phylogenetic analysis of phage CFP3 based on amino acid sequences of four different proteins. The phylogenetic analysis of phage CFP3 based on amino acid sequences of four different proteins, including (**A**) major capsid protein, (**B**) tape measure protein, (**C**) lysozyme protein, and (**D**) integrase protein.

The phylogenetic analysis of phage CFP3 is presented based on the amino acid sequences of four distinct proteins: the major capsid protein, tape measure protein, lysozyme protein, and integrase protein (Figure 5). In each subfigure, the branch lengths of the phylogenetic tree represent the evolutionary distances between sequences, and the numbers on the branches indicate bootstrap values, reflecting the support for each branch. The varying colors and sizes of the circles represent the magnitude of the bootstrap values, with darker colors and larger circles indicating higher support.

In Figure 5A, phage CFP3 clusters with *Haemophilus* phage HP1 and HP2, indicating a close evolutionary relationship and suggesting they share a common ancestor. Figure 5B shows phage CFP3 clustering with *Haemophilus* phage HP1, HP2, and *Pasteurella* phage F108, further underscoring their evolutionary relationship. In Figure 5C, CFP3 is again on the same branch as *Haemophilus* phage HP1 and HP2, demonstrating high similarity in the lysozyme protein sequences. Figure 5D reveals that CFP3 clusters with *Haemophilus* phage HP1, HP2, and *Pasteurella* phage F108, indicating high sequence similarity in their integrase proteins.

Overall, these results illustrate the phylogenetic relationships of phage CFP3 based on different protein sequences, further confirming its evolutionary links with *Haemophilus* phage HP1, HP2, and *Pasteurella* phage F108. This analysis provides valuable insights into the evolutionary history and functional characteristics of phage CFP3.

### 3.8. Genomic Synteny Analysis

**Figure 6:**
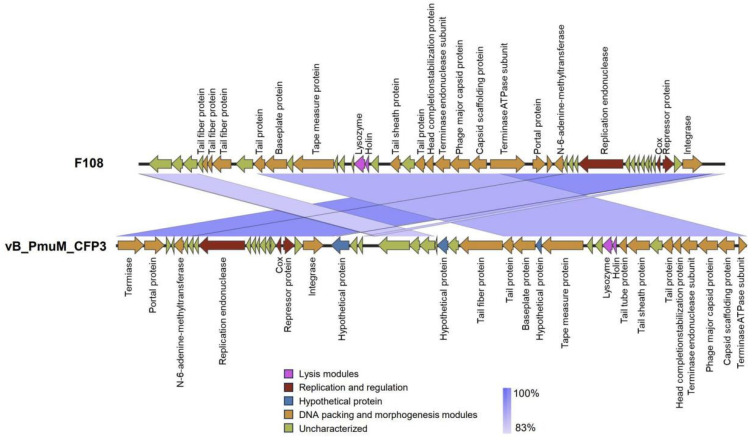
Comparative genomic synteny analysis between phage CFP3 and *Pasteurella* phage F108 at the nucleotide level. The gene organization of the two genomes is represented by arrows that indicate the transcriptional direction of genes. Different colored arrows represent different functional gene modules: pink arrows for the lysis module, red arrows for the replication and regulation module, blue arrows for hypothetical proteins, yellow arrows for DNA packaging and morphogenesis module, and green arrows for unclassified genes. Blue shaded areas indicate syntenic regions between the two genomes, with the shade intensity representing the percentage of synteny, where darker blue indicates 100% synteny and lighter blue indicates 83% synteny.

The genomic synteny comparison at the nucleotide level between phage CFP3 and *Pasteurella* phage F108 reveals that these two phages exhibit high synteny across multiple gene regions, particularly within the lysis module, replication and regulation module, and DNA packaging and morphogenesis module (Figure 6). This high degree of conservation in these functional regions suggests that the two phages may share a common ancestor. Additionally, the figure highlights that certain genes differ in position and orientation between the two genomes, indicating that gene rearrangement or horizontal gene transfer may have occurred during their evolution. Overall, this figure underscores the substantial genomic organizational similarity between phage CFP3 and *Pasteurella* phage F108, especially the conservation of key functional modules. This provides valuable insights into their evolutionary relationships and functional characteristics.

## 4. Discussion

This study successfully isolated and characterized a new phage, CFP3, from a chicken farm environment, providing an important foundation for developing alternative treatments for avian cholera. Through comprehensive biological characterization and genome sequencing, we gained an in-depth understanding of CFP3′s properties and its advantages and limitations as a potential therapeutic tool.

The successful isolation of CFP3 confirms the abundance of phage resources in the environment available for therapeutic purposes. The double-layer agar method combined with enrichment techniques not only effectively isolated a phage with high lytic activity but also provided a reliable methodological reference for future related research [17,18,19]. Transmission electron microscopy observation showed that CFP3 has a typical myovirus morphology, a structural feature similar to many known effective lytic phages, suggesting that CFP3 may have a good ability to infect and lyse host bacteria [20,21,22]. The size characteristics of a head diameter of about 60 nm and a tail length of about 120 nm also provide important references for future studies on the interaction mechanism between CFP3 and host bacteria.

The observed in vitro stability of CFP3 in the pH range of 5–10 and at temperatures up to 40 °C suggests its potential as a therapeutic candidate, though in vivo stability tests are necessary to confirm these findings. This stability allows CFP3 to adapt to different environments in the avian digestive tract, increasing its potential as an oral formulation. Meanwhile, temperature stability ensures the storage and transportation of CFP3 at room temperature, as well as its effectiveness at avian body temperature. These characteristics lay the foundation for the application of CFP3 in actual farming environments, but further in vivo experiments are still needed to verify its stability and effectiveness in living organisms.

Growth characteristic analysis showed that CFP3 has a latent period of about 90 min, which is relatively short, indicating that CFP3 can rapidly reproduce and effectively control bacterial growth. The finding of an optimal MOI of 1 has important guiding significance for the dose design of future phage preparations. However, it should be noted that the optimal MOI obtained under laboratory conditions may differ from actual application environments, so more factors need to be considered when developing actual treatment plans, such as the physiological state of the host animal and the method of administration [23,24,25].

CFP3′s lysis rate of 28.2% against avian-derived type A *P. multocida* reflects its relatively narrow host range. This specificity limits the widespread application of CFP3 but reduces the impact on non-target bacteria and minimizes interference with the host’s normal flora, making CFP3 a potential candidate for precision therapy, particularly suitable for *P. multocida* infections of specific strains. The narrow lysis spectrum of bacteriophages like CFP3 can be attributed to several factors, predominantly the genetic diversity among capsular groups of their bacterial hosts. This diversity significantly influences the ability of phages to effectively infect and lyse their target bacteria. Variations in capsular polysaccharides, critical for phage attachment, can create barriers to infection; even minor genetic differences can result in a phage being unable to recognize and bind to its host, thereby limiting its lytic activity [26].

Moreover, the evolutionary arms race between phages and bacteria, where bacteria evolve resistance mechanisms such as changes in surface receptors or protective biofilm production, forces phages to adapt. This coevolution often results in phages becoming specialized for a narrow range of bacterial strains, particularly if they have developed specific adaptations to overcome the defenses of certain capsular groups [27]. Additionally, the presence of temperate phages, which can integrate into the bacterial genome, further complicates lysis dynamics. These phages contribute to horizontal gene transfer among bacterial populations, potentially leading to increased genetic diversity and new capsular types not susceptible to existing phages [28]. 

Consequently, considering the possible existence of multiple *P. multocida* strains in actual farming environments, developing phage mixtures or seeking phages with a broader host range might be necessary to enhance therapeutic effects. The complex interactions between phages and their bacterial hosts, shaped by genetic diversity, evolutionary pressures, and temperate phages, profoundly influence the effectiveness of phage therapy and biocontrol strategies [29,30,31].

Genome analysis of CFP3 provided important information for understanding its biological characteristics and evolutionary relationships. The 32,696 bp linear double-stranded DNA genome, 41.7% G + C content, and 46 predicted ORFs reflect CFP3′s similarity to other known *Pasteurella* phages. The absence of tRNA genes and antibiotic-resistance genes in the genome reduces the risk of horizontal gene transfer, increasing the safety of CFP3 as a therapeutic agent. The 21 known functional protein-coding genes cover key functions required for the phage life cycle, indicating that CFP3 has the complete ability to infect and lyse hosts.

Phylogenetic analysis revealed a close evolutionary relationship between CFP3 and *Haemophilus* phages HP1, HP2, and *Pasteurella* phage F108. This relationship is reflected not only at the whole genome level but also in the amino acid sequences of major capsid protein, tape measure protein, lysozyme protein, and integrase protein. This finding provides important clues for understanding the origin and evolution of CFP3 while also suggesting that these related phages may have similar host ranges and infection mechanisms. Future research can explore the similarities and differences between CFP3 and related phages in terms of function and host adaptability based on this information, providing new ideas for optimizing phage therapy.

Genome synteny analysis further confirmed the high conservation of functional modules between CFP3 and *Pasteurella* phage F108, especially in the lysis module, replication and regulation module, and DNA packaging and morphogenesis module. This conservation not only reflects their common ancestor but also implies the importance of these functional modules in the phage life cycle. At the same time, differences in the position and direction of certain genes suggest that gene rearrangement or horizontal gene transfer may have occurred during evolution, which may be a mechanism for phages to adapt to different hosts and environments. An in-depth study of these differences may reveal the molecular basis of CFP3′s specific host range.

Although CFP3 has demonstrated several advantages as a potential therapeutic tool, there are still some challenges in developing it for practical applications. Firstly, the limited host range may restrict its widespread use, necessitating consideration of combining it with other phages or expanding its host range through genetic engineering. Secondly, although the possibility of phage-induced resistance is lower than that of antibiotics, vigilance against bacterial resistance to CFP3 is still needed. Conducting long-term resistance monitoring and studying bacterial resistance mechanisms to CFP3 will help formulate effective countermeasures.

Moreover, this study was mainly conducted under in vitro conditions, and the therapeutic effect and safety of CFP3 in vivo still need to be verified through animal experiments. This includes evaluating the effectiveness of different administration methods (such as oral or a spray), determining the optimal dose and frequency of administration, and monitoring the long-term effects on host health and gut microbiota. At the same time, it is necessary to develop efficient and stable phage production and purification processes to meet the needs of large-scale applications.

Based on the findings of this study, future research can be conducted in several aspects: First, expand the phage library by isolating and characterizing more phages targeting avian *P. multocida*, and develop effective phage mixtures to broaden the host range and reduce the risk of resistance development. Second, in-depth research on the molecular mechanisms of CFP3 infection and host lysis should be conducted to provide a theoretical basis for modifying and optimizing phages. Third, in vivo experiments will be conducted to evaluate the preventive and therapeutic effects of CFP3 in avian animal models, including studies on administration methods, dosage, and safety.

Furthermore, exploring the synergistic effects of CFP3 with other antimicrobial strategies (such as vaccines and probiotics) is also a promising research direction. This comprehensive approach may provide more effective strategies for avian cholera control. Meanwhile, based on CFP3′s genomic information, attempts can be made to enhance its lytic activity or expand its host range through genetic engineering, but this requires careful evaluation of potential biosafety risks.

## 5. Conclusions

In conclusion, this study successfully isolated and characterized phage CFP3, providing important basic data and theoretical support for developing alternative treatments for avian cholera. CFP3′s specific lytic activity, environmental adaptability, and genomic characteristics make it a promising candidate as a therapeutic tool. Despite facing some challenges, with further research and development of related technologies, CFP3 has the potential to become an effective means of controlling avian cholera and reducing antibiotic use. Future research should focus on evaluating the in vivo effects of CFP3, optimizing application strategies, and exploring its combined use with other control measures. As research on phage therapy advances and relevant regulations are improved, CFP3 and similar phage preparations are expected to provide safer and more effective bacterial infection control solutions for the poultry industry, making important contributions to sustainable agriculture and public health.

## Figures and Tables

**Table 1:** Determination of the optimal multiplicity of infection of the phage vB_PmuM_CFP3.

MOI(PFU/CFU)	Initial Phage Count (PFU)	Bacterial Count (CFU)	Final Phage Titer (PFU/mL)
10	2 × 10^9^	2 × 10^8^	(4.5 ± 0.7) × 10^8^
1	2 × 10^8^	2 × 10^8^	(6.9 ± 0.5) × 10^8^
0.1	2 × 10^7^	2 × 10^8^	(2.3 ± 0.3) × 10^8^
0.01	2 × 10^6^	2 × 10^8^	(3.5 ± 0.8) × 10^9^
0.001	2 × 10^5^	2 × 10^8^	(1.2 ± 0.9) × 10^8^
0.0001	2 × 10^4^	2 × 10^8^	(2.6 ± 0.9) × 10^7^

**Table 2:** The lysis spectrum of phage CFP3.

Strain	Source	Capsular Type	Lytic Ability
CVCC392	Cow	D	No plaque was observed
FCf79	Monkey	F	No plaque was observed
CVCC44801	Chicken	A	Plaque formation
FCf9	Duck	A	No plaque was observed
FCf12	Chicken	A	No plaque was observed
FCf20	Duck	A	Plaque formation
FCf25	Duck	A	No plaque was observed
FCf27	Duck	A	No plaque was observed
FCf33	Duck	A	No plaque was observed
FCf39	Goose	A	No plaque was observed
FCf45	Chicken	A	No plaque was observed
FCf58	Chicken	A	Plaque formation
FCf62	Duck	A	No plaque was observed
FCf64	Duck	A	No plaque was observed
FCf65	Chicken	A	Plaque formation
FCf70	Goose	A	No plaque was observed
FCf72	Duck	A	Plaque formation
FCf73	Duck	A	No plaque was observed
FCf85	Chicken	A	No plaque was observed
FCf86	Chicken	A	No plaque was observed
FCf87	Chicken	A	No plaque was observed
FCf88	Chicken	A	No plaque was observed
FCf93	Chicken	A	Plaque formation
FCf94	Duck	A	No plaque was observed
FCf95	Chicken	A	No plaque was observed
FCf91	Duck	A	Plaque formation
FCf102	Chicken	A	No plaque was observed
FCf103	Duck	A	Plaque formation
FCf104	Chicken	A	No plaque was observed
FCf105	Duck	A	No plaque was observed
FCf109	Goose	A	Plaque formation
FCf112	Chicken	A	No plaque was observed
FCf113	Goose	A	No plaque was observed
FCf114	Duck	A	Plaque formation
FCf115	Chicken	A	No plaque was observed
FCf126	Chicken	A	No plaque was observed
FCf128	Duck	A	Plaque formation
FCf134	Chicken	A	No plaque was observed
FCf135	Duck	A	No plaque was observed
FCf142	Goose	A	No plaque was observed
FCf143	Duck	A	No plaque was observed

**Table 3:** Functional annotation of ORFs genes of phage CFP3.

ORFs	Strand	Start	Stop	Identity (%)	Function	Best-Match BLASTp Result
1	+	85	1458	98.91	Terminase	Haemophilus phage HP1
2	+	1442	2494	76.76	Portal protein	Haemophilus phage HP1
3	+	2570	2845	/	/	/
4	−	2940	2842	/	/	/
5	−	3479	2937	96.25	N-6-adenine-methyltransferase	Pasteurella phage F108
6	−	3702	3448	/	/	/
7	−	3988	3716	/	/	/
8	−	4242	3991	/	/	/
9	−	6652	4256	99.87	Replication endonuclease	Pasteurella phage F108
10	−	6954	6682	/	/	/
11	−	7211	6960	/	/	/
12	−	7434	7225	/	/	/
13	−	7750	7415	/	/	/
14	−	7979	7743	/	/	/
15	+	8004	8246	/	/	/
16	−	8530	8318	90.74	Cox	Pasteurella phage F108
17	+	8655	9230	72.43	Repressor protein	Haemophilus phage S2
18	+	9230	9682	/	/	/
19	+	9685	10,707	70.41	Integrase	Haemophilus phage S2
20	−	12,073	11,147	/	/	/
21	−	12,501	12,076	/	/	/
22	−	12,758	12,519	/	/	/
23	−	15,177	13,576	/	/	/
24	−	15,746	15,177	/	/	/
25	−	16,497	15,733	/	/	/
26	−	16,607	16,476	/	/	/
27	−	17,167	16,607	/	/	/
28	−	17,745	17,143	/	/	/
29	−	20,017	17,756	46.15	Tail fiber protein	Haemophilus phage HP1
30	−	20,561	20,034	83.33	Tail protein	Haemophilus phage HP1
31	−	21,724	20,558	95.36	Baseplate protein	Pasteurella phage F108
32	−	22,055	21,717	/	/	/
33	−	24,182	22,059	97.17	Tape measure protein	Pasteurella phage F108
34	−	24,683	24,375	/	/	/
35	−	25,203	24,817	/	/	/
36	−	25,751	25,200	45.21	Lysozyme	Haemophilus phage HP1
37	−	25,950	25,738	91.43	Holin	Pasteurella phage F108
38	−	26,483	26,031	85.33	Tail tube protein	Pasteurella phage F108
39	−	27,618	26,488	78.46	Tail sheath protein	Haemophilus phage HP1
40	−	28,326	27,637	/	/	/
41	−	28,856	28,323	93.98	Tail protein	Pasteurella phage F108
42	−	29,305	28,853	68	Head completion/stabilization protein	Haemophilus phage HP1
43	−	30,144	29,278	84.58	Terminase endonuclease subunit	Pasteurella phage F108
44	−	31,172	30,165	72.21	Phage major capsid protein	Haemophilus phage HP1
45	−	32,063	31,176	85.42	Capsid scaffolding protein	Pasteurella phage F108
46	+	32,269	32,647	95.49	Terminase ATPase subunit	Pasteurella phage F108

Note: +—Normal strand; −—Minus strand; /—The ORFs that have not been annotated.

## Data Availability

The complete genome sequence of CFP3 has been deposited in GenBank under accession no. OQ025560.
